# Development and validation of a blood biomarker-based model for differentiating stroke etiology in acute large vessel occlusion

**DOI:** 10.3389/fneur.2025.1567348

**Published:** 2025-04-25

**Authors:** Weiwei Gao, Renjing Zhu, Jingjing She, Rong Huang, Lijuan Cai, Shouyue Jin, Yanping Lin, Jianzhong Lin, Xingyu Chen, Liangyi Chen

**Affiliations:** ^1^Department of Neurology, Zhongshan Hospital of Xiamen University, School of Medicine, National Advanced Center for Stroke, Xiamen Key Subspecialty of Neurointerventional Radiology, Xiamen University, Xiamen, China; ^2^Xiamen Clinical Research Center for Cerebrovascular Diseases, Xiamen, China; ^3^Xiamen Quality Control Center for Stroke, Xiamen, China; ^4^The School of Clinical Medicine, Fujian Medical University, Fuzhou, Fujian, China; ^5^School of Medicine, Xiamen University, Xiamen, China; ^6^Department of MRI, Zhongshan Hospital of Xiamen University, School of Medicine, Xiamen University, Xiamen, China

**Keywords:** large vessel occlusion, stroke etiology, cardioembolism, laboratory biomarkers, prediction model

## Abstract

**Objective:**

Early differentiation of stroke etiology in acute large vessel occlusion stroke (LVOS) is crucial for optimizing endovascular treatment strategies. This study aimed to develop and validate a prediction model for pre-procedural etiological differentiation based on admission laboratory parameters.

**Methods:**

We conducted a retrospective cohort study at a comprehensive stroke center, enrolling consecutive patients with acute LVOS who underwent endovascular treatment between January 2018 and October 2024. The study cohort (*N* = 415) was split into training (*n* = 291) and validation (*n* = 124) sets using a 7:3 ratio. We applied machine learning techniques—the Boruta algorithm followed by least absolute shrinkage and selection operator regression—for variable selection. The final predictive model was constructed using multivariable logistic regression. Model performance was evaluated through the area under the receiver operating characteristic curve (AUC), calibration plots, and decision curve analysis. We then developed a web-based calculator to facilitate clinical implementation.

**Results:**

Of 415 enrolled patients, 199 (48.0%) had cardioembolism (CE). The final model incorporated six independent predictors: age [adjusted odds ratio (aOR) 1.03], male sex (aOR 0.35), white blood cell count (aOR 0.86), platelet-large cell ratio (aOR 1.06), aspartate aminotransferase (aOR 1.02), and non-high-density lipoprotein cholesterol (aOR 0.75). The model demonstrated good discriminatory ability in both the training set (AUC = 0.802) and the validation set (AUC = 0.784). Decision curve analysis demonstrated consistent clinical benefit across threshold probabilities of 20%–75%.

**Conclusion:**

We developed and internally validated a practical model using routine admission laboratory parameters to differentiate between CE and large artery atherosclerosis in acute LVOS. This readily implementable tool could aid in preoperative decision-making for endovascular intervention.

## Introduction

Acute ischemic stroke remains a leading cause of global mortality and disability, claiming approximately 5 million lives annually (1). Large vessel occlusion stroke (LVOS), characterized by rapid clinical deterioration and poor outcomes, represents a particularly devastating subtype (2). Cardioembolism (CE) and large artery atherosclerosis (LAA) are the primary etiologies of LVOS, collectively accounting for 94.0% of all cases (3, 4). Endovascular thrombectomy has emerged as a crucial breakthrough in improving the outcomes of LVOS patients by achieving timely recanalization and salvaging the ischemic penumbra (5). However, the optimal endovascular treatment strategy varies depending on the underlying etiology. Although stent retrievers demonstrate similar initial recanalization rates in both etiologies, atherosclerotic occlusions often face the risk of re-occlusion due to *in situ* stenosis and platelet activation, frequently necessitating rescue treatments such as balloon angioplasty or stenting (6). Furthermore, direct aspiration techniques have shown significantly better efficacy in patients with CE compared to those with atherosclerotic lesions (7, 8). Therefore, accurate preoperative identification of the occlusion mechanism is crucial for determining the optimal treatment strategy.

However, current methods for etiological differentiation have limitations. Assessments based on baseline neuroimaging or preoperative angiographic features (e.g., presence of a stump, tapered occlusion, or truncal-type occlusion) heavily rely on operator experience (3, 9, 10), while predictive models based on cardiovascular risk factors and medical history are limited by potential underdiagnosis and variability in patient reporting (11). In contrast, admission laboratory examinations, as routinely required items before endovascular treatment, offer significant advantages in terms of universal accessibility, objectivity, and cost-effectiveness. Nevertheless, the potential of these parameters in predicting LVOS etiology has not been systematically explored.

We sought to develop and validate a predictive model using admission laboratory parameters to differentiate between CE and LAA in LVOS patients. These readily available biomarkers may reflect distinct pathophysiological processes and guide the selection of optimal endovascular strategies.

## Methods

### Study design and population

This study was a retrospective observational study based on an electronic medical record database. We consecutively enrolled patients with LVOS who underwent endovascular thrombectomy at Zhongshan Hospital of Xiamen University between January 2018 and October 2024. The study protocol was approved by the hospital’s ethics committee, which waived the requirement for informed consent due to the retrospective nature of the research.

The inclusion criteria were as follows: (1) age ≥18 years; (2) received thrombectomy treatment at our hospital within the appropriate time window (onset-to-puncture time ≤6 h for anterior circulation LVOS, or 6–24 h after onset but deemed suitable for endovascular treatment based on rigorous imaging evaluation; time window ≤24 h for posterior circulation LVOS); (3) presence of large vessel occlusion confirmed by computed tomography angiography or intraoperative digital subtraction angiography (DSA), with etiology classified as CE or LAA. This inclusion was justified because all blood samples were collected prior to any therapeutic interventions (including thrombolysis), and thrombolytic therapy does not significantly influence the differentiation of stroke etiology. Therefore, thrombolysis status was not considered an exclusion criterion. The exclusion criteria included: (1) large vessel occlusion caused by other etiologies, such as arterial dissection, hypercoagulable state, malignancy, hematological disorders, vasculitis, or vascular malformations (e.g., Moyamoya disease); (2) presence of severe cardiac, hepatic, or renal dysfunction, or hematological disorders; (3) incomplete emergency laboratory examinations or tests performed outside our hospital. The patient selection process is presented in Supplementary Figure 1.

### Sample size calculation

We calculated the sample size based on the events per variable criterion, which is a widely accepted method for statistical analysis. In our training set, the proportion of CE was 0.47. Considering our intention to include six predictive variables and setting the EPV at 10, we calculated the required sample size using the following formula:


$$\mathrm{Sample Size}=\frac{\mathrm{Number of Cariables}\times EPV}{1-\mathrm{Invidence Rate}}=\frac{6\times 10}{1-0.47}=113$$

### Data collection and laboratory analysis

We systematically collected demographic characteristics and results of emergency electrocardiographic examinations. All data were independently collected and recorded by two specially trained neurologists following a standardized protocol, and cross-checked by other researchers to ensure data accuracy and completeness.

Blood samples were collected immediately upon emergency admission and before any therapeutic interventions. All tests were performed in the central laboratory of our hospital using standardized methods and regularly calibrated automated analyzers, strictly adhering to the manufacturers’ operating instructions. To ensure the accuracy of the results, all blood samples were processed within 30 min of collection. Laboratory parameters included: (1) hematological parameters, measured using a fully automated hematology analyzer, including white blood cell series (total white blood cell count and counts and percentages of neutrophils, lymphocytes, and monocytes), red blood cell series (red blood cell count, hemoglobin, hematocrit, mean corpuscular volume, mean corpuscular hemoglobin, mean corpuscular hemoglobin concentration, and red cell distribution width), as well as platelet hematocrit, platelet distribution width, and platelet-large cell ratio; (2) coagulation function indicators, measured using an automated coagulation analyzer, including fibrinogen and D-dimer levels; (3) biochemical parameters, measured using a fully automated biochemical analyzer, including protein metabolism (total protein, albumin, globulin, albumin/globulin ratio), liver function (alanine aminotransferase, aspartate aminotransferase), lipid profile (triglycerides, total cholesterol, high-density lipoprotein cholesterol, low-density lipoprotein cholesterol), and renal function (blood urea nitrogen, creatinine, uric acid).

### Calculation of composite biomarkers

Based on existing evidence suggesting that composite biomarkers may have more stable and accurate predictive value than single indicators, we calculated the following composite indicators based on routine laboratory test results and divided them into three categories:

1. Inflammation-related indicators:

Neutrophil-to-lymphocyte ratio (NLR) = neutrophil count / lymphocyte count.

Systemic inflammation response index (SIRI) = (neutrophil count × monocyte count) / lymphocyte count.

1. Metabolism-related indicators:

Non-high-density lipoprotein cholesterol (Non-HDL cholesterol) = total cholesterol (mmol/L) − high-density lipoprotein cholesterol (mmol/L).

Non-high-density lipoprotein cholesterol to high-density lipoprotein cholesterol ratio (NHHR) = Non-HDL-C (mmol/L) / HDL-C (mmol/L).

Hemoglobin to red blood cell distribution width ratio (HRR) = (hemoglobin (g/L) × 0.1) / red cell distribution width coefficient of variation (%).

Triglyceride-glucose index (TyG) = ln[(triglycerides (mmol/L) × 88.57) × (glucose (mmol/L) × 18.0156) / 2].

1. Organ function-related indicators:

Blood urea nitrogen-to-albumin ratio (BAR, mg/g) = (urea (mmol/L) × 2.801) / (albumin (g/L) × 0.1).

### Stroke etiology classification

The determination of stroke etiology was based on a comprehensive assessment of clinical characteristics, risk factors, auxiliary examination results, and findings during endovascular treatment. All patients underwent a thorough etiological evaluation during hospitalization, including detailed history taking, cardiovascular risk factor assessment (hypertension and diabetes), and systematic diagnostic investigations (emergency electrocardiography, 24-h Holter monitoring, carotid ultrasound, right heart contrast echocardiography, transthoracic echocardiography, and bilateral lower extremity venous ultrasound).

According to TOAST criteria (11, 12) and considering the findings during endovascular treatment, we classified patients into the LAA group and the CE group. The diagnostic criteria for LAA were as follows: digital subtraction angiography immediately after thrombectomy showed significant stenosis (>50% or tendency for re-occlusion after successful reperfusion) in the responsible vessel, with corresponding atherosclerotic changes confirmed by CT or MR angiography, while excluding high-risk sources of cardioembolism. The diagnostic criteria for CE were as follows: complete recanalization after the thrombectomy, no evidence of atherosclerosis, and the presence of a definite high-risk source of cardioembolism, including mechanical valves, mitral stenosis with atrial fibrillation, atrial fibrillation (except lone atrial fibrillation), left atrial/left atrial appendage thrombus, sick sinus syndrome, recent myocardial infarction (<4 weeks), left ventricular thrombus, or patent foramen ovale with atrial septal aneurysm. For patients with suspected cardioembolism, repeated electrocardiographic monitoring was performed postoperatively to detect potential paroxysmal atrial fibrillation.

Patients with multiple etiologies or unclear etiology were excluded from the study. The final etiological classification of all patients was jointly assessed by an attending physician and two interventional radiologists, with consensus reached through team discussion in case of disagreement.

### Statistical analysis

Categorical variables were described as frequencies (percentages) [*n* (%)], and comparisons between groups were performed using Pearson’s chi-square test or Fisher’s exact test. The normality of continuous variables was assessed using the Shapiro–Wilk test. Normally distributed variables were presented as mean ± standard deviation, and comparisons between groups were performed using the independent samples t-test; non-normally distributed variables were presented as median (interquartile range), and comparisons between groups were performed using the Mann–Whitney U test.

The study cohort was randomly divided into a training set (*n* = 291) and a validation set (*n* = 124) at a 7:3 ratio. In the training set, we first used the Boruta algorithm for high-dimensional data screening to preliminarily determine potential predictive variables. To avoid overfitting and multicollinearity issues, we further optimized the variable selection process based on the initial screening results using least absolute shrinkage and selection operator (LASSO) regression. Finally, we used a multivariable logistic regression model to identify independent risk factors and construct a nomogram for predicting the etiology of acute LVOS. To facilitate the application of the model in clinical practice, we developed a web-based interactive nomogram tool using the Shiny package in R.

The discriminative ability of the predictive model was assessed using receiver operating characteristic (ROC) curves, and the area under the ROC curve (AUC) and its 95% confidence interval (CI) were reported. The AUC ranges from 0.5 (no discrimination) to 1.0 (perfect discrimination). The calibration ability of the model was evaluated using calibration plots, which assess the accuracy of the model by comparing the predicted probabilities with the actual observed probabilities. We also used decision curve analysis (DCA) to evaluate the clinical net benefit of the model at different threshold probabilities. Statistical analyses were performed using two-sided tests, and the significance level was set at *α* = 0.05. All statistical analyses were conducted using R software (Version 4.2.2, R Foundation for Statistical Computing, Vienna, Austria).

## Results

### Demographics and baseline characteristics

A total of 415 patients with LVOS who received MT treatment were ultimately included in this study (Table 1). Patients were randomly allocated to the training cohort (*n* = 291) and internal test cohort (*n* = 124) at a 7:3 ratio. Among all patients, 214 (51.82%) were classified as LAA, and 199 (48.18%) as CE. Of these, 128 (30.99%) patients had atrial fibrillation on admission emergency electrocardiography, but 46% of CE patients had no obvious abnormalities on admission electrocardiography. The training and test cohorts showed no significant differences in baseline characteristics such as age [67 (57–75) years vs. 70 (59–78) years, *p* = 0.119], sex (male 70.1% vs. 62.1%, *p* = 0.110), stroke etiology (LAA 53.26% vs. 48.36%, *p* = 0.363), and laboratory parameters (Table 1). Overall, the baseline characteristics were evenly distributed between the training and internal test cohort, supporting the validity of the predictive model established in this study.

**Table 1:** Baseline clinical, and laboratory characteristics of patients with emergent large vessel occlusion stroke in the training and internal test cohorts.

Variables	Overall	Training cohort	Internal test cohort	*p*-value
	*N* = 415	*N* = 291	*N* = 124	
Age, years	68 (58, 76)	67 (57, 75)	70 (59, 78)	0.119
Sex, male	281 (67.7)	204 (70.1)	77 (62.1)	0.110
Stroke etiology				0.363
Large-artery atherosclerosis	214 (51.82)	155 (53.26)	59 (48.36)	
Cardioembolism	199 (48.18)	136 (46.74)	63 (51.64)	
Positive emergency ECG findings	128 (30.99)	83 (28.52)	45 (36.89)	0.094
Complete blood count				
White blood cell, ×10^9^/L	8.6 (6.6, 11.4)	8.6 (6.7, 11.2)	8.3 (6.3, 11.9)	0.678
Neutrophils count, ×10^9^/L	6.0 (4.3, 9.0)	6.1 (4.4, 9.0)	5.7 (4.2, 8.8)	0.586
Lymphocytes count, ×10^9^/L	1.48 (1.02, 2.23)	1.45 (1.00, 2.20)	1.50 (1.07, 2.28)	0.606
Monocytes, ×10^9^/L	0.46 (0.34, 0.61)	0.47 (0.36, 0.61)	0.43 (0.32, 0.62)	0.328
Neutrophil Percentage, %	74 (63, 83)	74 (63, 83)	74 (64, 83)	0.921
Lymphocyte Percentage, %	18 (12, 28)	18 (12, 28)	18 (12, 28)	0.817
Red blood cells, ×10^12^/L	4.55 (4.13, 4.97)	4.56 (4.14, 5.00)	4.47 (4.10, 4.86)	0.233
Hemoglobin, g/L	139 (124, 150)	139 (124, 150)	138 (126, 148)	0.688
Hematocrit, %	41.4 (38.3, 44.6)	41.7 (38.5, 44.8)	41.2 (38.0, 43.9)	0.533
MCV, fL	91.2 (87.9, 95.2)	91.1 (87.8, 94.9)	91.9 (88.7, 95.5)	0.208
MCH, pg	30.60 (29.35, 31.90)	30.40 (29.20, 31.80)	30.80 (29.50, 31.90)	0.153
MCHC, g/L	334 (325, 343)	333 (325, 341)	335 (326, 343)	0.360
RDW-CV, %	13.10 (12.50, 13.95)	13.10 (12.60, 14.00)	13.00 (12.48, 13.73)	0.375
Platelet distribution width, fL	11.00 (9.70, 12.80)	11.00 (9.70, 12.95)	10.70 (9.70, 12.63)	0.581
Platelet-large cell ratio, %	23 (19, 29)	23 (19, 29)	22 (19, 28)	0.584
Biochemical parameters				
Total protein, g/L	72 (68, 75)	72 (68, 76)	71 (68, 75)	0.319
Albumin, g/L	40.2 (37.6, 42.4)	40.4 (37.6, 42.6)	39.6 (37.3, 42.1)	0.263
Globulin, g/L	31.5 (28.5, 34.4)	31.9 (28.4, 34.4)	30.8 (28.8, 34.5)	0.698
Albumin to globulin ratio	1.28 ± 0.20	1.28 ± 0.20	1.27 ± 0.20	0.579
Triglycerides, mmol/L	1.23 (0.86, 1.82)	1.25 (0.86, 1.77)	1.20 (0.85, 1.90)	0.969
Total cholesterol, mmol/L	4.73 (3.93, 5.47)	4.73 (3.89, 5.47)	4.72 (4.11, 5.45)	0.527
HDL cholesterol, mmol/L	1.15 (0.99, 1.39)	1.15 (0.97, 1.37)	1.17 (1.03, 1.45)	0.073
LDL cholesterol, mmol/L	3.07 (2.49, 3.68)	3.04 (2.45, 3.70)	3.13 (2.62, 3.60)	0.490
Alanine aminotransferase, U/L	16 (11, 23)	17 (12, 24)	15 (11, 20)	0.112
Aspartate aminotransferase, U/L	23 (19, 30)	23 (19, 30)	23 (19, 30)	0.445
Blood urea nitrogen, mmol/L	6.00 (4.80, 7.12)	5.90 (4.65, 7.02)	6.13 (5.03, 7.37)	0.281
Creatinine, μmol/L	74 (61, 91)	74 (61, 91)	73 (63, 91)	0.785
Uric acid, μmol/L	390 (322, 455)	394 (321, 456)	381 (325, 445)	0.505
Coagulation parameters				
D-dimer, mg/L FEU	1.4 (0.5, 2.9)	1.5 (0.5, 2.8)	1.4 (0.5, 3.2)	0.880
Fibrinogen, g/L	3.06 (2.62, 3.66)	3.05 (2.63, 3.58)	3.08 (2.62, 3.82)	0.576
Composite biomarkers				
NLR	4.1 (2.3, 7.0)	4.1 (2.3, 7.1)	4.1 (2.2, 6.8)	0.809
SIRI	1.8 (1.0, 3.3)	1.8 (1.0, 3.4)	1.6 (0.9, 2.9)	0.332
HRR	1.06 (0.92, 1.17)	1.06 (0.92, 1.17)	1.04 (0.94, 1.15)	0.958
NHHR	2.95 (2.15, 3.83)	2.97 (2.14, 3.89)	2.84 (2.15, 3.69)	0.720
Non-HDL cholesterol, mmol/L	3.47 (2.73, 4.18)	3.44 (2.70, 4.20)	3.50 (2.87, 4.14)	0.650
Triglyceride-glucose index	10.37 (9.98, 10.75)	10.37 (9.98, 10.73)	10.30 (9.97, 10.76)	0.999
BAR, mg/g	4.20 (3.32, 5.13)	4.14 (3.22, 5.08)	4.33 (3.48, 5.40)	0.236

Values are presented as median (interquartile range) or mean ± standard deviation, as appropriate. MCV, mean corpuscular volume; MCH, mean corpuscular hemoglobin; MCHC, mean corpuscular hemoglobin concentration; RDW-CV, red blood cell distribution width coefficient of variation; HDL, high-density lipoprotein; LDL, low-density lipoprotein; NLR, neutrophil-to-lymphocyte ratio; SIRI, systemic inflammation response index; HRR, hemoglobin-to-red cell distribution width ratio; NHHR, non-HDL-to-HDL cholesterol ratio; BAR, blood urea-to-albumin ratio; FEU, fibrinogen equivalent units.

### Comparison of laboratory parameters in the training cohort

In the emergency admission laboratory examinations of the training cohort (Table 2), CE patients exhibited significantly different demographic and biomarker characteristics compared to the LAA group. CE patients were older [72 (62–80) vs. 63 (55–70) years, *p* < 0.001] and had a higher proportion of females (42.6% vs. 18.7%, *p* < 0.001). Emergency hematological tests showed lower levels of inflammatory markers in the CE group, as evidenced by lower white blood cell count [7.8 (6.3–9.7) × 10^9^/L vs. 9.4 (7.5–12.9) × 10^9^/L, *p* < 0.001] and neutrophil count [5.2 (3.9–7.4) × 10^9^/L vs. 6.6 (5.1–11.0) × 10^9^/L, *p* < 0.001]. Moreover, CE patients had significantly lower hemoglobin levels [135 (122–145) vs. 143 (129–154) g/L, *p* < 0.001] but markedly higher platelet-large cell ratio [26% (20–31%) vs. 22% (18–27%), *p* < 0.001].

**Table 2:** Comparison of baseline laboratory parameters between large-artery atherosclerosis and cardioembolism groups in the training cohort of patients with large vessel occlusion stroke.

Variables	LAA group (*n* = 155)	CE group (*n* = 136)	*p* value
Age, years	63 (55, 70)	72 (62, 80)	<0.001
Sex, male	126 (81.3)	78 (57.4)	<0.001
Complete blood count			
White blood cell count, ×10^9^/L	9.4 (7.5, 12.9)	7.8 (6.3, 9.7)	<0.001
Neutrophils count, ×10^9^/L	6.6 (5.1, 11.0)	5.2 (3.9, 7.4)	<0.001
Lymphocytes count, ×10^9^/L	1.50 (1.05, 2.35)	1.42 (0.98, 2.00)	0.248
Monocytes count, ×10^9^/L	0.51 (0.38, 0.64)	0.43 (0.34, 0.58)	0.008
Neutrophil Percentage, %	76 (66, 84)	70 (61, 83)	0.056
Lymphocyte Percentage, %	17 (10, 26)	21 (12, 29)	0.064
Red blood cells, ×10^12^/L	4.71 (4.28, 5.12)	4.44 (4.06, 4.87)	0.001
Hemoglobin, g/L	143 (129, 154)	135 (122, 145)	<0.001
Hematocrit, %	42.3 (39.0, 46.2)	40.5 (36.8, 43.4)	0.002
Mean corpuscular volume, fL	90 (88, 94)	93 (89, 96)	0.008
Mean corpuscular hemoglobin, pg	30.30 (29.30, 31.75)	30.90 (29.10, 31.83)	0.383
MCHC, g/L	335 (327, 344)	330 (324, 338)	0.002
RDW-CV, %	13.00 (12.50, 13.60)	13.20 (12.68, 14.33)	0.013
Platelet distribution width, fL	10.70 (9.50, 12.30)	11.60 (10.08, 13.23)	0.001
Platelet-large cell ratio, %	22 (18, 27)	26 (20, 31)	<0.001
Biochemical parameters			
Total protein, g/L	72 (68, 76)	72 (68, 76)	0.615
Albumin, g/L	40.4 (37.6, 42.6)	40.6 (38.4, 43.2)	0.020
Globulin, g/L	31.9 (28.4, 34.4)	31.4 (28.6, 33.9)	0.230
Albumin to globulin ratio	1.27 (1.18, 1.39)	1.30 (1.19, 1.40)	0.615
Triglycerides, mmol/L	1.35 (1.00, 1.85)	1.13 (0.79, 1.60)	0.004
Total cholesterol, mmol/L	5.04 (4.21, 5.80)	4.45 (3.49, 5.14)	<0.001
HDL cholesterol, mmol/L	1.13 (0.98, 1.35)	1.17 (0.96, 1.39)	0.870
LDL cholesterol, mmol/L	3.29 ± 0.92	2.85 ± 0.94	<0.001
Alanine aminotransferase, U/L	16 (12, 23)	18 (12, 27)	0.075
Aspartate aminotransferase, U/L	22 (18, 26)	26 (21, 33)	<0.001
Blood urea nitrogen, mmol/L	5.50 (4.60, 6.60)	6.41 (5.10, 7.81)	<0.001
Creatinine, μmol/L	73 (62, 91)	76 (60, 91)	0.690
Uric acid, μmol/L	389 (320, 450)	402 (325, 464)	0.253
Coagulation parameters			
D-dimer, mg/L FEU	3.16 (2.71, 3.60)	3.02 (2.59, 3.54)	0.294
Fibrinogen, g/L	1.0 (0.4, 2.6)	1.7 (0.9, 3.2)	<0.001
Composite biomarkers			
NLR	4.4 (2.6, 8.2)	3.4 (2.2, 6.6)	0.058
SIRI	2.06 (1.16, 4.16)	1.48 (0.91, 2.84)	0.003
HRR	1.11 (0.95, 1.21)	1.01 (0.86, 1.13)	<0.001
NHHR	3.26 (2.49, 3.99)	2.61 (1.91, 3.46)	<0.001
Non-HDL cholesterol, mmol/L	3.84 (3.01, 4.48)	3.23 (2.41, 3.79)	<0.001
Triglyceride-glucose index	10.46 (10.03, 10.80)	10.26 (9.92, 10.62)	0.013
BAR, mg/g	3.66 (3.04, 4.71)	4.55 (3.60, 5.90)	<0.001

Values are presented as median (interquartile range) unless otherwise specified; mean ± standard deviation is used for normally distributed variables. LAA, large-artery atherosclerosis; CE, cardioembolism; MCHC, mean corpuscular hemoglobin concentration; RDW-CV, red blood cell distribution width coefficient of variation; HDL, high-density lipoprotein; LDL, low-density lipoprotein; NLR, neutrophil-to-lymphocyte ratio; SIRI, systemic inflammation response index; HRR, hemoglobin-to-red cell distribution width ratio; NHHR, non-HDL-to-HDL cholesterol ratio; BAR, blood urea-to-albumin ratio.

Biochemical tests at admission revealed that CE patients generally had lower lipid levels, including total cholesterol [4.45 (3.49–5.14) mmol/L vs. 5.04 (4.21–5.80) mmol/L, *p* < 0.001], LDL cholesterol (2.85 ± 0.94 mmol/L vs. 3.29 ± 0.92 mmol/L, *p* < 0.001), and non-HDL cholesterol [3.23 (2.41–3.79) mmol/L vs. 3.84 (3.01–4.48) mmol/L, *p* < 0.001]. Characteristic changes in the CE group also included significantly elevated blood urea nitrogen levels [6.41 (5.10–7.81) mmol/L vs. 5.50 (4.60–6.60) mmol/L, *p* < 0.001] and aspartate aminotransferase levels [26 (21–33) U/L vs. 22 (18–26) U/L, *p* < 0.001].

Regarding composite biomarkers, the CE group exhibited lower systemic inflammation response index (SIRI: 1.48 vs. 2.06, *p* = 0.003) and non-HDL cholesterol to HDL cholesterol ratio (NHHR: 2.61 vs. 3.26, *p* < 0.001) but higher blood urea nitrogen to albumin ratio [4.55 (3.60–5.90) vs. 3.66 (3.04–4.71) mg/g, *p* < 0.001].

### Variable selection for the prediction model

**Figure 1:**
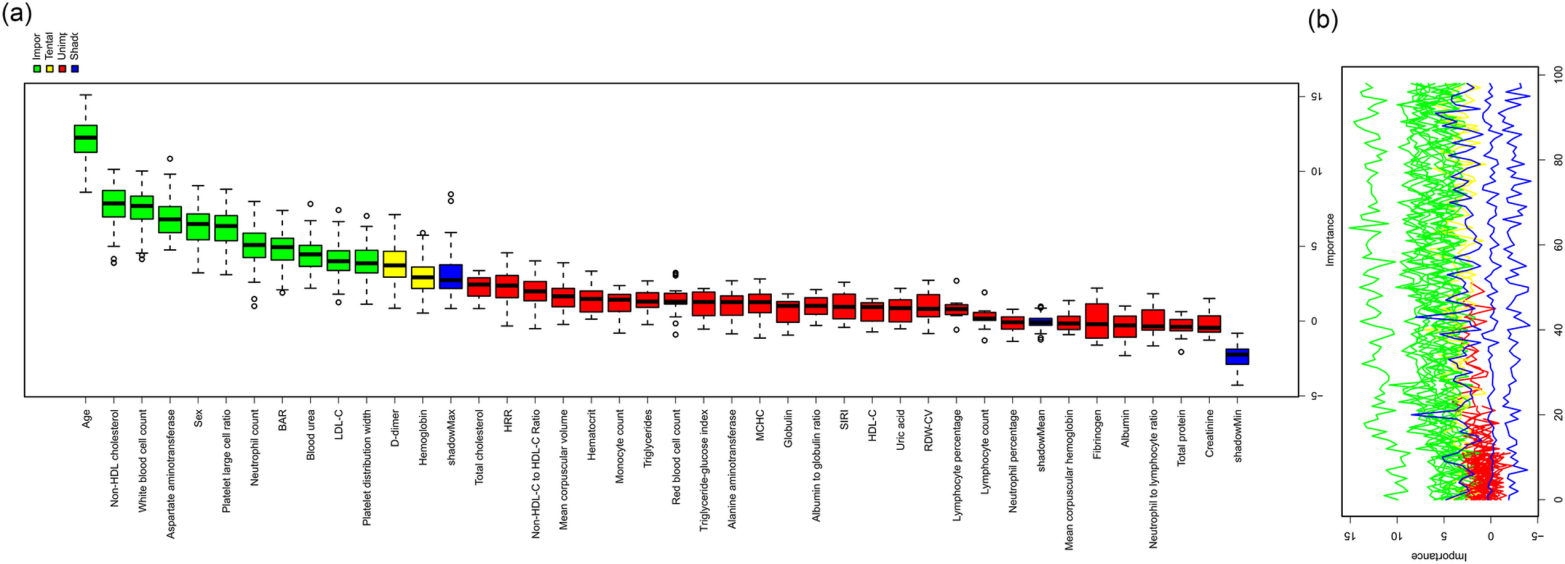
Variable importance analysis using the Boruta algorithm for LVOS etiology prediction. **(a)** Boxplot showing the relative importance of laboratory parameters and composite biomarkers based on Boruta algorithm analysis. Green boxes represent confirmed important variables, yellow boxes represent tentatively important variables, and red boxes represent rejected variables. **(b)** Time series plot showing the convergence of importance scores over 100 iterations of the Boruta algorithm for confirmed (green), tentative (yellow), and rejected (red) variables.

To construct a robust predictive model, we employed the Boruta algorithm, a feature selection method based on random forest, to assess the importance of 40 candidate variables (Figure 1). With the maximum number of iterations set to 100, the algorithm ultimately identified 13 important predictive variables: age, sex, inflammatory status (white blood cell count, neutrophil count), lipid parameters (total cholesterol, low-density lipoprotein cholesterol, non-high-density lipoprotein cholesterol), platelet-large cell ratio, platelet distribution width, blood urea nitrogen, aspartate aminotransferase, blood urea nitrogen to albumin ratio, and D-dimer. Additionally, two variables (hemoglobin and hemoglobin to red blood cell distribution width ratio) were marked as tentatively important, requiring further evaluation. The remaining 25 variables did not demonstrate significant predictive value.

**Figure 2:**
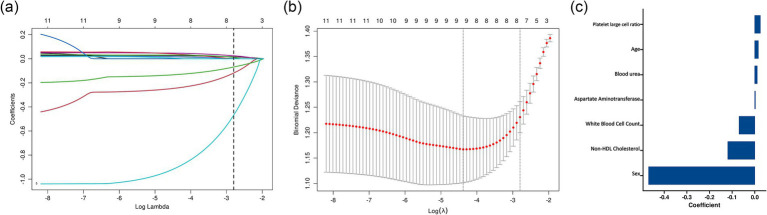
LASSO regression analysis for variable selection in the prediction model. **(a)** LASSO coefficient profiles of the candidate variables plotted against the log(*λ*) values. Each colored line represents a variable’s coefficient path. The vertical dashed line represents the optimal λ value selected through cross-validation. **(b)** Cross-validation error curve showing the binomial deviance (±1 SE) against log(λ). **(c)** Bar plot showing the standardized coefficients of the six variables selected by LASSO regression at the optimal λ value. Variables are ordered by the absolute magnitude of their coefficients, with sex showing the strongest association.

To further optimize the prediction model and avoid overfitting and multicollinearity, we performed least absolute shrinkage and selection operator (LASSO) regression analysis on the 13 variables selected by the Boruta algorithm (Figure 2). The model was trained using 10-fold cross-validation and 100 candidate *λ* values. At the optimal λ value, seven key predictive variables were finally determined: age, sex, white blood cell count, platelet-large cell ratio, aspartate aminotransferase, blood urea nitrogen, and non-high-density lipoprotein cholesterol.

### Logistic regression analysis and model development

Univariate and multivariate logistic regression analyses were performed on the seven variables selected by LASSO regression (Table 3). Univariate analysis showed that all variables were significantly associated with cardioembolism (all *p* < 0.01). In the multivariate model, six independent predictors maintained statistical significance: increasing age (adjusted odds ratio [aOR] 1.03, 95% confidence interval [CI] 1.01–1.05), elevated platelet-large cell ratio (aOR 1.06, 95% CI 1.02–1.10), and higher aspartate aminotransferase levels (aOR 1.02, 95% CI 1.00–1.04) were significantly associated with an increased risk of CE (all *p* < 0.05). Conversely, male sex (aOR 0.35, 95% CI 0.19–0.63, *p* < 0.001), higher white blood cell count (aOR 0.86, 95% CI 0.79–0.93, *p* < 0.001), and elevated non-high-density lipoprotein cholesterol (aOR 0.75, 95% CI 0.59–0.95, *p* = 0.017) were associated with a reduced risk of CE. Blood urea nitrogen lost statistical significance after adjusting for other factors (aOR 1.08, 95% CI 0.96–1.21, *p* = 0.201).

**Table 3:** Univariate and multivariate logistic regression analysis for predicting cardioembolism in patients with large vessel occlusion stroke.

Variables	Univariate analysis		Multivariate analysis	
	OR (95% CI)	*p* value	OR (95% CI)	*p* value
Age	1.05 (1.03, 1.07)	<0.001	1.03 (1.01, 1.05)	0.014
Sex, male	0.31 (0.18, 0.52)	<0.001	0.35 (0.19, 0.63)	<0.001
White blood cell	0.84 (0.78, 0.90)	<0.001	0.86 (0.79, 0.93)	<0.001
Platelet-Large Cell Ratio	1.07 (1.03, 1.11)	<0.001	1.06 (1.02, 1.10)	0.006
Aspartate aminotransferase	1.03 (1.01, 1.04)	0.003	1.02 (1.00, 1.04)	0.020
Blood urea nitrogen	1.19 (1.07, 1.32)	0.001	1.08 (0.96, 1.21)	0.201
Non-HDL cholesterol	0.64 (0.51, 0.79)	<0.001	0.75 (0.59, 0.95)	0.017

Values are presented as odds ratio (OR) with 95% confidence interval (CI).

**Figure 3:**
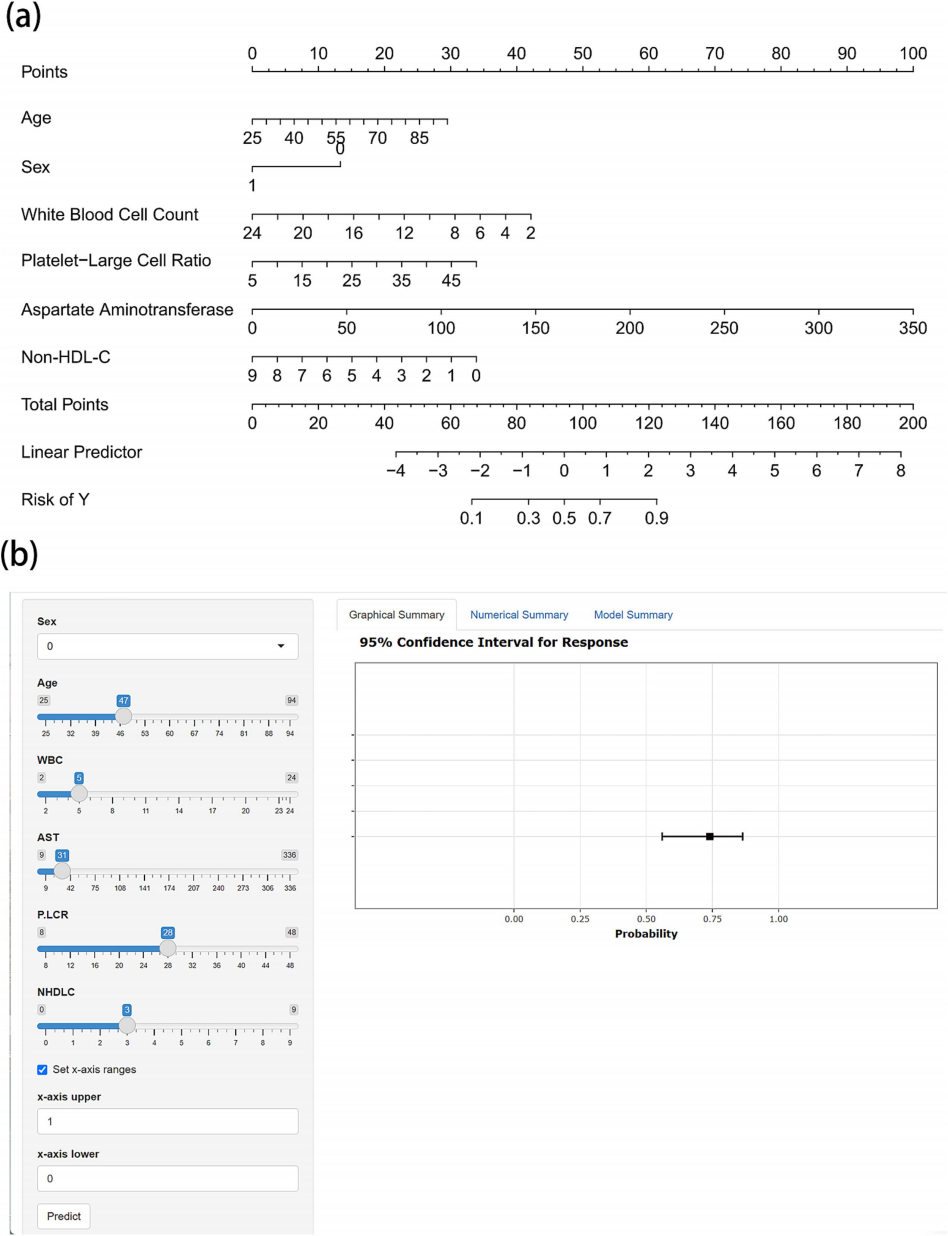
Nomogram and web-based calculator for predicting cardioembolism in acute LVOS patients. **(a)** Nomogram for estimating the probability of cardioembolism. Points are assigned for each variable by drawing a vertical line from the variable value to the “Points” line. The sum of points plotted on the “Total Points” line corresponds to the predicted probability of cardioembolism on the “Risk of Y” line. **(b)** Screenshot of the web-based interactive calculator (available online at https://gaoww.shinyapps.io/dynnomapp/). The tool provides real-time probability estimates with 95% confidence intervals based on input laboratory values. The interface allows for rapid clinical assessment through slider-based input and immediate visual feedback.

Based on these independent predictors, we developed a nomogram for assessing the risk of cardioembolism in patients with acute LVOS (Figure 3). To facilitate clinical application, we also constructed a web-based interactive predictive tool,^1^ enabling rapid assessment of individualized risk for patients.

### Model performance and clinical utility assessment

**Figure 4:**
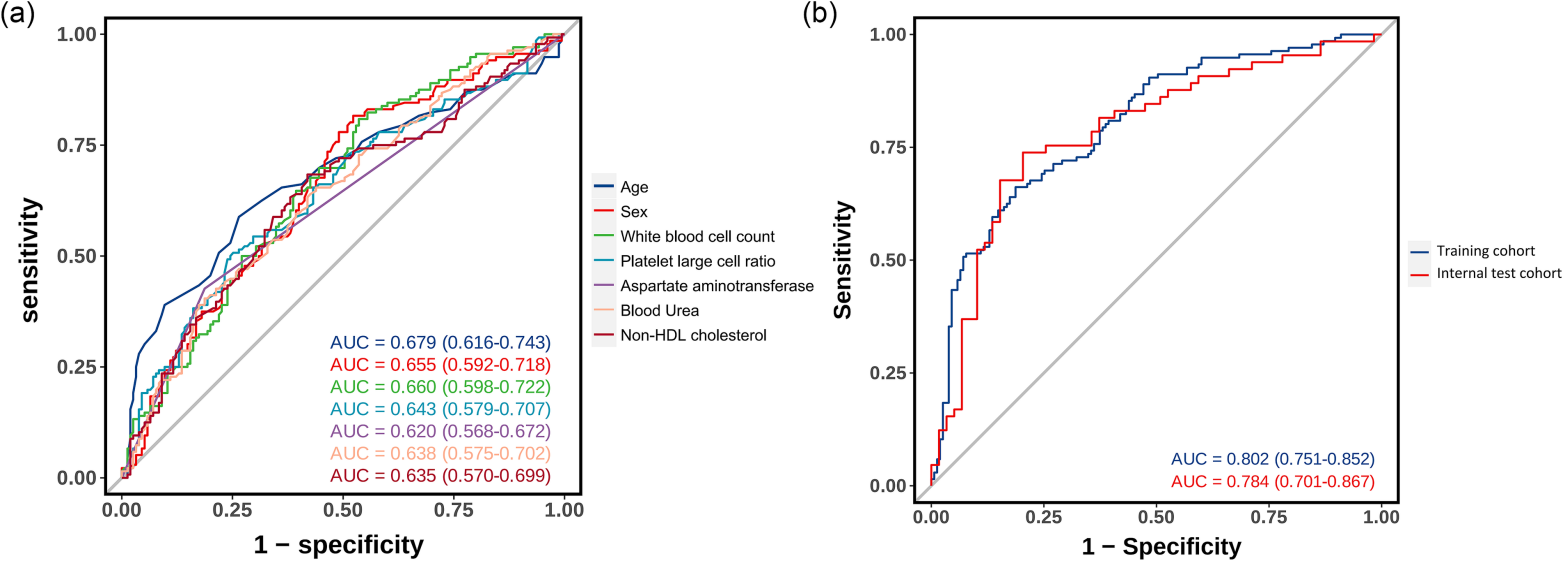
Receiver operating characteristic curves showing the discriminative performance of predictive variables and the integrated model. **(a)** ROC curves for individual predictive variables. **(b)** ROC curves comparing model performance in training and internal validation cohorts.

The predictive model demonstrated good discriminatory ability in the training cohort (AUC = 0.802, 95% CI 0.751–0.852), which was validated in the internal validation cohort (AUC = 0.784, 95% CI 0.701–0.867) (Figure 4). ROC curve analysis of individual predictive variables showed that age had the highest discriminatory power (AUC = 0.679, 95% CI 0.616–0.743), followed by sex (AUC = 0.655, 95% CI 0.592–0.718) and white blood cell count (AUC = 0.660, 95% CI 0.598–0.722).

**Figure 5:**
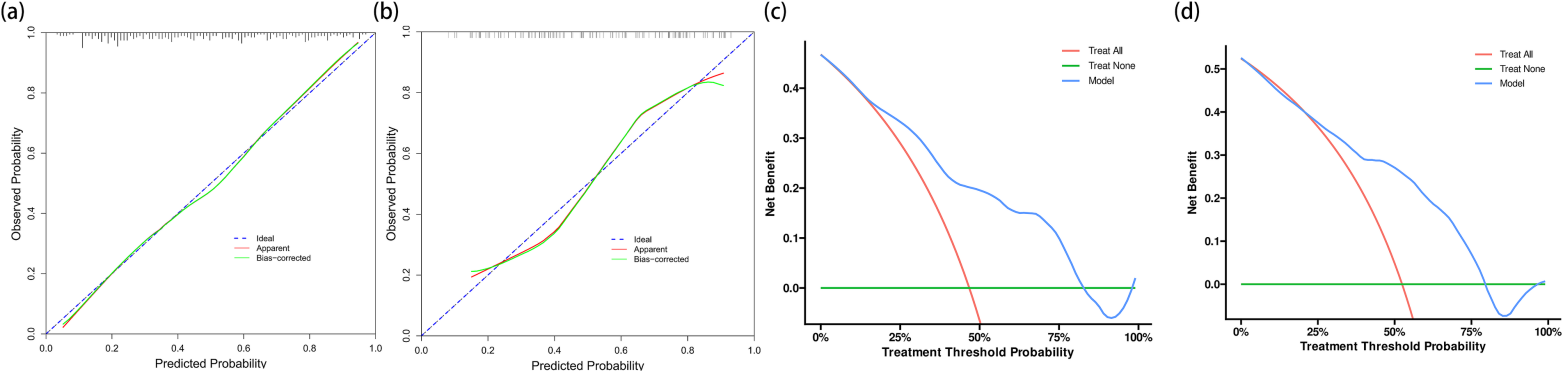
Calibration plots and decision curve analyses for model evaluation in training and validation cohorts. **(a)** Calibration plot for the training cohort (*n* = 291) showing excellent agreement between predicted and observed probabilities of cardioembolism. **(b)** Calibration plot for the internal validation cohort (*n* = 124). Although showing slight deviation at extreme probabilities, the model maintains good calibration across most of the probability range. **(c)** Decision curve analysis for the training cohort demonstrating the net benefit of the prediction model (blue line) compared to the strategies of treating all patients as CE (red line) or none as CE (green line). The model shows clinical utility across threshold probabilities of 20%–75%. **(d)** Decision curve analysis for the internal validation cohort confirming the model’s clinical utility, with consistent net benefit patterns observed in the training cohort.

Calibration plot assessment (Figure 5) revealed that the probabilities predicted by the model exhibited good consistency with the observed outcomes, a characteristic that was evident in both the training and internal validation cohorts. The calibration curve of the training cohort almost perfectly aligned with the ideal curve, while the internal validation cohort showed only slight deviations in the extreme ranges of predicted probabilities, indicating stable predictive accuracy of the model.

DCA further verified the clinical utility of the predictive model (Figure 5). The results showed that using the model to predict CE yielded significant net benefits within the threshold probability range of 20%–75%, demonstrating clear advantages over the strategies of “assuming all patients have CE” or “assuming all patients have non-CE.” This finding was consistently validated in the internal validation cohort.

## Discussion

In this retrospective observational study, we found that 46% of patients ultimately diagnosed with CE had no obvious abnormalities on admission electrocardiography. Given the crucial importance of etiological differentiation in optimizing endovascular treatment strategies for LVOS, accurately identifying CE patients with negative electrocardiographic findings and those with LAA is of significant clinical relevance. Using machine learning methods, we developed and validated a predictive model based on readily available admission laboratory parameters. We identified six independent predictors: age, sex, white blood cell count, platelet-large cell ratio, aspartate aminotransferase, and non-high-density lipoprotein cholesterol. This integrated approach demonstrated robust discriminatory ability in both the training set (AUC = 0.802) and the internal validation set (AUC = 0.784), outperforming the predictive efficacy of any single indicator. Furthermore, we revealed characteristic laboratory manifestations of CE, including a higher proportion of females, older age, attenuated inflammatory response, and lower lipid levels. Decision curve analysis showed that the model had significant clinical utility within a wide range of threshold probabilities (20%–75%).

Our findings are consistent with previous research, which has shown that patients with LVOS caused by CE are younger and have a higher proportion of males compared to those with LAA (13). Although these two subtypes exhibit characteristic differences in clinical presentation—CE often presents with sudden onset and rapid progression, while atherosclerotic stroke tends to manifest as progressive worsening and is frequently accompanied by a history of transient ischemic attacks—these clinical features are often difficult to accurately ascertain in the emergency setting. Previous studies have attempted to differentiate stroke etiology from multiple perspectives. Angiographic features have shown some value, with Jin et al. (14) finding that a jet-like appearance is a specific imaging marker of atherosclerotic occlusion, and Yi et al. (15) confirming the significant diagnostic value of the microcatheter first-pass effect (90.9% vs. 12.8%, *p* < 0.001). However, these features can only be confirmed during endovascular intervention and are difficult to guide preoperative decision-making. The development of clinical prediction tools has also made some progress, such as the scoring system constructed by Liao et al. (11) that integrates atrial fibrillation, blood pressure, neurological deficits, CT findings, and diabetes. However, this method is limited by multiple factors, including potential underdiagnosis of cardiovascular risk factors, differences in population health literacy, and the inability of patients with neurological deficits to accurately provide medical history. Moreover, the inclusion of complications discovered during hospitalization may not accurately reflect the preoperative state. Recently, Li et al. (16) conducted a study from a metabolomics perspective, constructing a predictive model based on triglycerides and sphingolipids that demonstrated excellent discriminatory ability (AUC = 0.889). Despite its superior performance, the complexity and high cost of metabolomics analysis limit its application in clinical practice, especially in primary healthcare settings. As a routinely required item before endovascular treatment, emergency laboratory examinations offer significant advantages, including universal accessibility, strong objectivity, and low cost. However, no studies have systematically explored the feasibility of constructing predictive models based on emergency laboratory indicators.

The different laboratory characteristics observed in our study may reflect the underlying pathophysiological differences between CE and LAA. The higher incidence of CE in elderly female patients is consistent with the increased prevalence of atrial fibrillation in this population, aligning with the established understanding of age and female sex as recognized risk factors for arrhythmia (17). In terms of lipid profiles, LAA patients exhibited significantly elevated levels of non-high-density lipoprotein cholesterol, which encompasses both remnant cholesterol and low-density lipoprotein cholesterol. Previous research has demonstrated that elevated remnant cholesterol levels can accelerate cholesterol accumulation within the arterial wall, promoting the progression of atherosclerosis and leading to cardiovascular events (18). Furthermore, studies have confirmed that elevated non-high-density lipoprotein cholesterol is a crucial determinant of culprit lesion plaque burden in acute coronary syndrome (19), directly correlating with the necrotic core volume of atherosclerotic plaques and exhibiting a stronger association with the progression of coronary atherosclerosis compared to low-density lipoprotein cholesterol (19, 20). These findings validate our study conclusions. Our analysis revealed that non-high-density lipoprotein cholesterol demonstrated superior predictive value compared to low-density lipoprotein cholesterol in differentiating CE from LAA.

Inflammatory markers also showed significant differences between the two stroke subtypes. Patients with atherosclerotic stroke exhibited a more pronounced low-grade inflammatory state (21). This process involves complex interactions between lipid abnormalities and reduced cholesterol efflux, promoting the production of mononuclear cells in the hematopoietic system. Simultaneously, oxidized low-density lipoprotein triggers the release of epigenetically modified monocytes, which can sustain ongoing inflammatory responses. These phenotypically altered monocytes and macrophages exhibit adaptive immune responses rather than innate immune behavior, maintaining a persistent inflammatory state. In contrast, CE primarily originates from the acute detachment of intracardiac thrombi, with a lower degree of inflammatory involvement, which is consistent with our observation of lower white blood cell and monocyte counts in the CE group.

Furthermore, we found that higher aspartate aminotransferase levels were significantly associated with an increased risk of CE. However, the exact link between aspartate aminotransferase and AF remains unclear. Sinner et al. (22) reported that elevated ALT and AST concentrations were associated with an increased incidence of AF over a 10-year follow-up period (HR: 1.12, 95% CI: 1.01–1.24, *p* = 0.03). Nevertheless, two other prospective cohort studies failed to confirm this association (23, 24). Elevated aspartate aminotransferase levels may indicate subtle myocardial injury, as this enzyme is also present in cardiac tissue and can be released under conditions of mild myocardial stress. This finding could be either a cause or a consequence of cardioembolic events, warranting further investigation.

We observed that the CE group exhibited a significantly increased platelet-large cell ratio, suggesting enhanced platelet turnover and activation (25). Physiological studies have shown that platelets have a lifespan of 3–6 days (26). Moreover, previous research has found that hemodynamic changes caused by underlying heart diseases, such as valvular heart disease, can lead to chronic platelet stress and activation (27). In contrast, atherosclerotic occlusion is usually secondary to acute plaque rupture, where platelet activation is a secondary response to exposed subendothelial components. The acute nature of atherosclerotic events may not be sufficient to induce significant changes in platelet production and volume distribution. This temporal dynamics characteristic is consistent with the differences in platelet features observed between the two stroke subtypes. However, the underlying mechanisms warrant further exploration.

This study has several limitations. First, the retrospective single-center study design may introduce selection bias. Second, although the model performed well in internal validation, external validation in different populations and healthcare settings is still required to confirm its generalizability. Third, our strategic decision to rely solely on laboratory parameters has both methodological advantages and limitations. This approach ensured standardization and objectivity, minimizing variability and reporting bias in clinical assessments. However, it may fail to capture all clinically relevant information. We acknowledge that the reporting and recording of cardiovascular risk factors can vary significantly between different healthcare institutions due to differences in health literacy, diagnostic capabilities, and documentation standards. For example, the diagnosis rates of atrial fibrillation, hypertension, and diabetes may differ substantially between tertiary hospitals and primary care facilities, or between urban and rural areas. Although our model based on laboratory tests maintains broad applicability, it may sacrifice some potential predictive power from comprehensive clinical information. Future research should consider developing region-specific models that integrate laboratory parameters with standardized clinical assessments, including emergency electrocardiography results and validated stroke scales. Such comprehensive models, calibrated according to local healthcare capabilities and population characteristics, may achieve higher predictive accuracy while maintaining practicality in specific healthcare settings.

## Conclusion

We developed and internally validated a practical model using routine admission laboratory parameters to differentiate between CE and LAA in acute LVOS. The model’s robust discrimination and established clinical utility suggest its potential value in guiding endovascular intervention strategies. Beyond its predictive capability, our findings revealed distinct laboratory patterns between stroke subtypes, providing novel insights into their underlying pathophysiological mechanisms. This laboratory-based approach offers a readily implementable tool for rapid etiological assessment in emergency settings, particularly valuable when electrocardiographic findings are inconclusive. External validation across diverse populations and healthcare settings is warranted to confirm these findings and establish the model’s broader clinical applicability.

## Data Availability

The datasets presented in this article are not readily available because the datasets used and/or analyzed during the current study are available from the corresponding author upon reasonable request. Requests to access the datasets should be directed to LCh, chenliangyizsyy@126.com.

## Footnotes

^1^ https://gaoww.shinyapps.io/dynnomapp/

## Supplementary material

SUPPLEMENTARY FIGURE 1 CONSORT flow diagram of patient selection and allocation. Flow diagram showing the patient selection process. From 535 initially screened patients with large vessel occlusion stroke who underwent endovascular thrombectomy, 120 were excluded based on predefined criteria. The remaining 415 patients were randomly allocated in a 7:3 ratio to the training cohort (*n* = 291) and validation cohort (*n* = 124), with cardioembolism (CE) and large artery atherosclerosis (LAA) distribution as indicated.
