# Supplementary figures and images for: Development and validation of a blood biomarker-based model for differentiating stroke etiology in acute large vessel occlusion
Source: Front Neurol. 2025 Apr 25;16:1567348. doi: 10.3389/fneur.2025.1567348 (PMC12061931; doi:10.3389/fneur.2025.1567348)

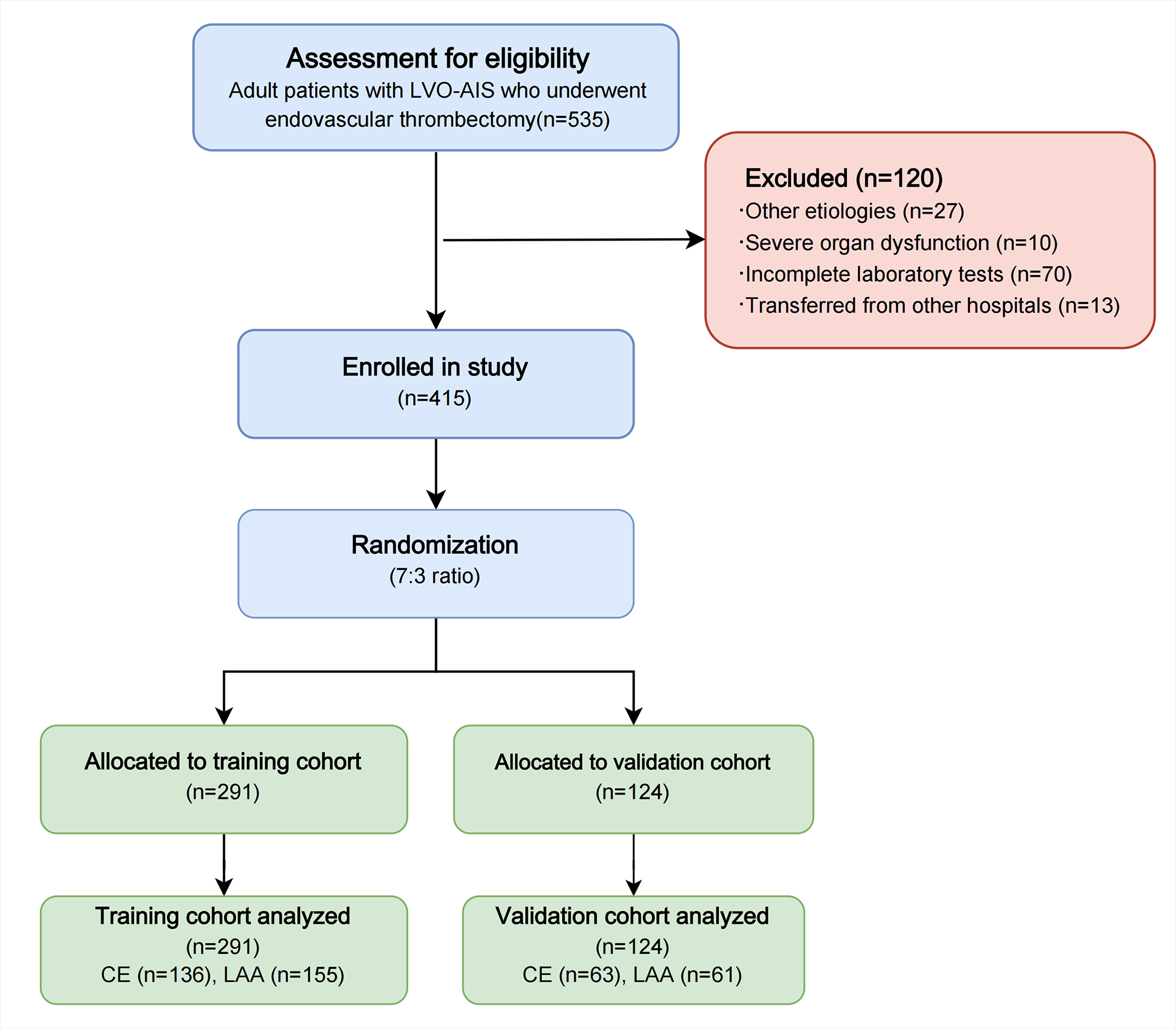

Supplement: SUPPLEMENTARY FIGURE 1 — CONSORT flow diagram of patient selection and allocation. Flow diagram showing the patient selection process. From 535 initially screened patients with large vessel occlusion stroke who underwent endovascular thrombectomy, 120 were excluded based on predefined criteria. The remaining 415 patients were randomly allocated in a 7:3 ratio to the training cohort (n = 291) and validation cohort (n = 124), with cardioembolism (CE) and large artery atherosclerosis (LAA) distribution as indicated. [file Image_1.TIF]
